# Impact of Health Interventions on Patient Compliance and Clinical Outcomes in Individuals With Diabetes Mellitus

**DOI:** 10.7759/cureus.84958

**Published:** 2025-05-28

**Authors:** Said Malook, Hassan Parvaiz, Javeria Sahani, Muhammad Arbi, Wajeeh Ur Rehman, Muniba Alam, Mahpara Laiq, Rabia Mehboob

**Affiliations:** 1 Emergency Medicine, Peshawar General Hospital, Peshawar, PAK; 2 Medicine, Abdullah Hospital, Lalamusa, PAK; 3 Internal Medicine, Aga Khan University Hospital, Karachi, PAK; 4 Medicine, Bahawalpur College of Pharmacy, Bahawalpur, PAK; 5 Internal Medicine, Saidu Group of Teaching Hospitals, Swat, PAK; 6 Medicine, Bahauddin Zakariya University, Multan, PAK; 7 Pharmacy, COMSATS University Islamabad, Lahore, PAK; 8 Medicine, CMH Medical College, Bahawalpur, PAK

**Keywords:** diabetes self-management questionnaire, health care outcomes, lifestyle intervention, technology innovations in health industry, value in health care

## Abstract

Introduction

Diabetes is a major cause of worldwide morbidity and mortality. Diet and medication non-adherence are common among individuals with diabetes, making glycemic control difficult to attain.

Objective

To assess the effect of a personalized health intervention on treatment compliance and clinical outcomes in patients with diabetes mellitus (DM).

Methodology

This quasi-experimental study was conducted at the Aga Khan University Hospital, Karachi, from January 2023 to December 2023. Data were collected from 250 DM patients who had constant and direct access to telecommunication devices. Participants were randomly assigned to two groups: Group A, the intervention group, and Group B, the control group. Patient compliance was assessed using a medication adherence questionnaire, which included questions about physical activity and medical appointments.

Results

Data were collected from 250 diabetic patients in two groups. The mean age in the intervention group was 55.01 ± 9.81 years and in the control group 57.23 ± 9.25 years. There were 125 participants in each group, and among these, in the intervention group, 48% were male and 52% were female. In the intervention group, HbA1c levels decreased from 8.5 ± 1.2% at baseline to 7.2 ± 1.0% at follow-up, compared to marginal changes in the control group (8.7 ± 1.1% to 8.4 ± 1.0%). In the intervention group, mean scores for physical functioning increased from 65 ± 5 at baseline to 75 ± 6 at follow-up, indicating enhanced functional capacity. Similarly, mental health scores showed improvement, with mean values rising from 70 ± 7 to 75 ± 8. Emotional well-being also demonstrated significant enhancement, with mean scores increasing from 60 ± 6 to 70 ± 7.

Conclusion

It is concluded that the health intervention implemented in this study significantly improved patient compliance and positively impacted health outcomes in diabetes management. With higher adherence rates to prescribed treatments and substantial improvements in key health metrics, including HbA1c levels, blood pressure, and cholesterol, the intervention demonstrated its efficacy.

## Introduction

Diabetes mellitus (DM) is a chronic metabolic problem characterized by raised blood glucose levels, and has emerged as a worldwide health challenge of stunning extent. With an expected 463 million grown-ups impacted overall starting around 2019, diabetes presents a significant burden on healthcare systems and social orders at large. Among these intricacies, cardiovascular infection stands apart as a main source of morbidity and mortality among people with diabetes [1]. Health interventions intended to improve patient consistency have arisen as vital methodologies in relieving the burden of diabetes and working on understanding health [2]. In any case, the adequacy of these interventions remains variable, with various elements affecting their outcomes. Routine medical care begins with planning for the patient with the supplier for preventive care services [3]. The patient can be booked for the following visit following a supplier visit or sometime in the future when the patient demands an arrangement by telephone or electronically. Interventions that proactively plan for the patient with their supplier are needed for opportune treatment options [4]. Whenever patients are booked for their supplier arrangements, the next step is to ensure that they attend their appointments. Concentrates on showing that flake-out rates for diabetic patients change from 4 to 40% [5]. Writing additionally demonstrates that diabetic patients with higher flake-out rates have more unfortunate outcomes, e.g., higher glycosylated hemoglobin (HbA1c) levels and less favorable glycemic control than patients who adhere to arrangements [6]. The quantity of digital health interventions (DHIs) conveyed through gadgets, for example, cell phones, tablets, or PCs has expanded dramatically [7]. Such DHIs have been utilized to work with remote access to powerful therapies, work on the management of chronic conditions, and promote healthy behaviors. Past systematic surveys have provided evidence about the impact of DHIs on health outcomes and patient experience [8]. Nonetheless, the financial impact of DHIs is less well-known. From one viewpoint, one could anticipate that DHIs should be cost-saving since they might assist with conveying healthcare more proficiently [9]. Then again, the execution of DHIs might suggest huge speculations by the health system, for instance, rebuilding care pathways and health administrations, execution expenses, and staff training and upskilling. While financial proof is rapidly emerging, the general course and magnitude of the monetary impact of DHIs is unclear. Health literacy is the crossing point of general literacy, health, and healthcare, yet it can likewise integrate components of different sorts of literacy to different degrees [10]. The conviction that individuals require something other than broad literacy abilities to deal with the intricacy of health and health system issues led to the concept of health literacy. There is a critical overlay between broad literacy and health literacy. In any case, there are unmistakable health-specific requests in health literacy that contrast from those of overall literacy [11]. In other words, having general literacy alone is insufficient if one has any desire to carry on with a healthy lifestyle and have the option to avoid, make due, and control sicknesses and diseases. Various examinations have shown that an absence of health literacy evaluation leads to healthcare experts misjudging clients' health literacy abilities [12].

This study aimed to evaluate the effectiveness of a structured telehealth intervention on improving treatment adherence and clinical outcomes, including HbA1c, blood pressure, lipid profile, and quality of life, among patients with type 2 diabetes.

## Materials and methods

Methodology

This quasi-experimental study was conducted at Aga Khan University Hospital Karachi from January 2023 to December 2023. A total of 250 diabetic patients were selected based on specific inclusion and exclusion criteria. Ethical approval for the study was obtained from the Institutional Review Board (IRB) of Aga Khan University Hospital, Karachi, prior to the commencement of data collection. We calculated the number of participants needed to see possible differences in HbA1c between the intervention and control groups. Based on previous studies evaluating the impact of health interventions on glycemic control, the use of health interventions indicated a 1.0% difference in HbA1c and a standard deviation of 2.0%, which are both signs of clinical significance. Applying a two-tailed t-test with α = 0.05 and 1-β = 80%, the calculation gave us the need for 98 participants per group. Since the dropout rate could reach 20%, the researchers adjusted the target to include 125 participants per group and a sample size of 250 patients.

The inclusion criteria for participants were age greater than 18 years, willingness to participate in the study, constant access to mobile phones and email for communication, and readiness for follow-up assessments. Patients were not included if they were diagnosed with type 1 DM, gestational diabetes, had serious psychiatric, cognitive, or neurological disorders, or if they had major illnesses that would result in frequent needs for hospitalization, such as end-stage renal disease or active malignancy. Patients who were unwilling to participate in the study or had any clinical or psychiatric disorder other than DM were excluded. 

Data collection

Data were collected from 250 DM patients who had constant and direct access to telecommunication devices. Participants were randomly assigned to two groups, Group A (intervention group) and Group B (control group). Patients in Group A were receiving personalized health education, dietary counseling, and exercise recommendations. This personalized approach aimed to improve patient self-management of diabetes through regular communication and feedback via mobile devices and email. Patients in Group B were receiving standard care, which did not include personalized health education or regular follow-up interventions. Group A consisted of patients who were receiving personalized health education, dietary counseling, and exercise recommendations. The health education intervention used in this study was developed in consultation with endocrinologists, diabetes educators, and public health specialists to ensure relevance, accuracy, and cultural appropriateness. These interventions aimed to improve patient self-management of diabetes through tailored communication and regular follow-ups, using mobile phones and email for support. Group B, the control group, consisted of patients who were receiving standard care, which did not include personalized health education or follow-up interventions, providing a comparison group for the study. Patient compliance was a key variable in this study and was assessed through self-reported adherence to medication, dietary guidelines, and exercise routines. A medication adherence questionnaire was used, which also included questions about physical activity levels and medical appointment attendance. This questionnaire served as an effective tool for understanding how well patients followed their prescribed treatment regimens. A questionnaire was designed to check patient compliance, with questions about sticking to medication, eating, exercising, and keeping medical appointments. Previously proven adherence scales for managing chronic diseases, such as the Morisky Medication Adherence Scale (MMAS-8) and the Summary of Diabetes Self-Care Activities (SDSCA), were used to create this new instrument. For content validity, the questionnaire was assessed by six experts working in diabetes, clinical pharmacy, and public health education. Once approved by experts, the questionnaire was tested on 20 diabetes patients not involved in the main study. The team checked responses to determine how harmonious and clear they were. The Cronbach’s alpha value of 0.87 demonstrates that the items are highly consistent internally. In addition to compliance data, baseline demographic information, such as age, disease history, medication use, and socioeconomic status, was also recorded for each participant. These variables helped to account for potential confounders in the analysis of health outcomes.

Data analysis

Data were analyzed using SPSS (IBM SPSS Statistics for Windows, IBM Corp., Version 26, Armonk, NY). Descriptive statistics, age, and gender were measured in mean ± SD. Independent t-tests and paired t-tests were applied to compare clinical outcomes and compliance measures within and between groups. Chi-square tests were used for categorical variables. A p-value of <0.05 was considered statistically significant.

## Results

Data were collected from 250 diabetic patients in two groups. The mean age in the intervention group was 55.01 ± 9.81 years and in the control group 57.23 ± 9.25 years. There were 125 participants in each group; among these, in the intervention group, 60 (48%) were male and 65 (52%) were female. Hypertension was present in 80 participants (64%) in the intervention group and 75 (60%) in the control group; dyslipidemia in 60 (48%) vs. 55 (44%); and cardiovascular disease in 30 (24%) vs. 35 (28%). Most patients were either overweight or obese, with 40 (32%) in the overweight and 25 (20%) in the obese category for both groups. A majority of participants were non-smokers, 75 (60%) in the intervention group and 70 (56%) in the control group (Table 1).

**Table 1:** Demographic data of participants in both groups

Demographic Variable	Intervention Group (n = 125)	Control Group (n = 125)
Age (years)		
- Mean ± SD	55.01 ± 9.81	57.23 ± 9.25
Gender		
- Male	60 (48%)	65 (52%)
- Female	65 (52%)	60 (48%)
Education		
- College Education	50 (40%)	45 (36%)
- High School Diploma	45 (36%)	50 (40%)
- Less Than High School Education	30 (24%)	30 (24%)
Occupation		
- Professional	40 (32%)	35 (28%)
- Blue Collar	45 (36%)	40 (32%)
- Unemployed/Retired	40 (32%)	50 (40%)
Comorbidities		
- Hypertension	80 (64%)	75 (60%)
- Dyslipidemia	60 (48%)	55 (44%)
- Cardiovascular Disease	30 (24%)	35 (28%)
Duration of Diabetes		
- <5 years	40	35
- 5-10 years	30	25
- >10 years	30	40
Body Mass Index (BMI)		
- Normal (18.5-24.9 kg/m²)	35	30
- Overweight (25-29.9 kg/m²)	40	45
- Obese (≥30 kg/m²)	25	25
Smoking Status		
- Non-smoker	75	70
- Former Smoker	15	20
- Current Smoker	10	10
Family History of Diabetes		
- Yes	60	55
- No	40	45

After a 12-week follow-up, the intervention group showed significant improvements in key clinical outcomes compared to the control group. HbA1c levels decreased from 8.5 ± 1.2% to 7.2 ± 1.0% (p < 0.001), while the control group showed only a slight reduction. Systolic blood pressure dropped from 135 ± 10 mmHg to 130 ± 8 mmHg (p = 0.032), and significant reductions were also observed in total cholesterol, low-density lipoprotein (LDL), and triglycerides (Table 2).

**Table 2:** Comparison of clinical outcomes between intervention and control groups at baseline and 12-week follow-up Significant p-values are denoted by an asterisk (*).

Outcome	Intervention Group (Baseline)	Intervention Group (Follow-up)	Control Group (Baseline)	Control Group (Follow-up)	t-Value	p-Value
HbA1c (%)	8.5 ± 1.2	7.2 ± 1.0	8.7 ± 1.1	8.4 ± 1.0	4.32	<0.001*
Systolic BP (mmHg)	135 ± 10	130 ± 8	138 ± 12	133 ± 10	2.15	0.032*
Diastolic BP (mmHg)	80 ± 6	78 ± 5	82 ± 7	80 ± 6	1.87	0.065
Total Cholesterol (mg/dL)	200 ± 20	180 ± 15	205 ± 22	200 ± 20	3.45	0.001*
Low-Density Lipoprotein (LDL) Cholesterol (mg/dL)	120 ± 15	110 ± 12	125 ± 18	120 ± 15	2.78	0.007*
Triglycerides (mg/dL)	150 ± 25	130 ± 20	155 ± 30	150 ± 25	3.12	0.003*
BMI (kg/m²)	30 ± 2	29 ± 2	31 ± 3	30 ± 3	1.68	0.097

In the intervention group, mean scores for physical functioning increased from 65 ± 5 at baseline to 75 ± 6 at follow-up, indicating enhanced functional capacity. Similarly, mental health scores showed improvement, with mean values rising from 70 ± 7 to 75 ± 8. Emotional well-being also demonstrated significant enhancement, with mean scores increasing from 60 ± 6 to 70 ± 7 (Table 3).

**Table 3:** Quality of life scores at baseline and follow-up Data have been presented as mean ± SD.

Domain	Intervention Group (Baseline: Mean ± SD)	Intervention Group (Follow-up: Mean ± SD)	Control Group (Baseline: Mean ± SD)	Control Group (Follow-up: Mean ± SD)
Physical Functioning	65 ± 5	75 ± 6	63 ± 4	65 ± 5
Mental Health	70 ± 7	75 ± 8	68 ± 6	70 ± 7
Emotional Well-Being	60 ± 6	70 ± 7	58 ± 5	60 ± 6

In the control group, medication adherence increased from 80% at baseline to 85% at follow-up, while dietary adherence improved from 70% to 75%. Moreover, attendance at appointments significantly rose from 90% to 95%. Conversely, the control group showed more modest changes, with medication adherence increasing from 60% to 65%, dietary adherence from 50% to 55%, and attendance at appointments from 75% to 80% (Table 4).

**Table 4:** Patients’ compliance at baseline and follow-up

Measure	Intervention Group (Baseline) (%)	Intervention Group (Follow-up) (%)	Control Group (Baseline) (%)	Control Group (Follow-up) (%)
Medication Adherence	80	85	60	65
Dietary Adherence	70	75	50	55
Attendance at Appointments	90	95	75	80

## Discussion

DM remains a worldwide health concern, described by its chronicity, different difficulties, and requiring broad regimens. The administration of diabetes frequently requires lifestyle modifications, medication adherence, and customary medical follow-ups, which present huge difficulties to patients' overall consistency and eventually impact health outcomes [13]. Ji et al.'s subjective examination as a feature of a similar report finds that the more limited the duration of diabetes since diagnosis, the more noteworthy the probability of finishing an intervention, and thus the higher the expenses caused [14]. Significant upgrades in persistence, especially in medication and dietary adherence, were noted in the intervention group compared to the control group. These progressions were joined by great health outcomes, including a significant reduction in HbA1c levels, improved blood pressure control, and positive modifications in lipid profiles and BMI [15,16]. The noticed contrasts among gauge and follow-up values highlight the adequacy of the health intervention in improving diabetes management. Contrasting these discoveries and existing literature, our review lines up with past examinations showing that fitted health interventions can prompt better quality outcomes in diabetes care [17]. In any case, while steady for certain examinations, certain errors in our outcomes warrant further examination. For example, the unobtrusive changes in specific health outcomes in the control group feature the possible impact of standard care practices and the requirement for complete intervention strategies [18]. Health literacy is imperative to guarantee access to care, taking care of oneself in ongoing circumstances, and support of health and prosperity; it is furthermore fundamental for healthcare, empowering people to assume a larger part in direction and management [19]. Medication consistency is connected to health literacy. When contrasted with patients with sufficient health literacy, patients with low health literacy have been shown to have less information about how to accept their medications as recommended. There are some study limitations also, first, this is a single-center study with a limited number of patients. Thus, we cannot generalize this study to the whole population. Since the study only included people who frequently use digital devices, socioeconomically or technology-challenged individuals may have been excluded. Also, since the participants self-reported on how well they followed the medication, diet, and appointment plans, they might have exaggerated their compliance because of poor memories or a wish to be accepted. Naturally, if participants and researchers are not masked, there is a risk that their involvement could impact participants’ actions as well as the review of results.

## Conclusions

It is concluded that the health intervention implemented in this study significantly improved patient compliance and had a positive impact on health outcomes in diabetes management. The intervention group demonstrated substantial reductions in HbA1c, systolic blood pressure, and lipid levels, along with improved adherence to medication, diet, and scheduled appointments. These findings support the integration of low-cost, patient-centered, and technology-assisted approaches into standard diabetes care.
